# Molecular Basis of the Toxigenic Vibrio cholerae O1 Serotype Switch from Ogawa to Inaba in Haiti

**DOI:** 10.1128/spectrum.03624-22

**Published:** 2022-12-20

**Authors:** Taylor K. Paisie, Melanie N. Cash, Massimiliano S. Tagliamonte, Afsar Ali, J. Glenn Morris, Marco Salemi, Carla Mavian

**Affiliations:** a Department of Pathology, Immunology and Laboratory Medicine, University of Florida, Gainesville, Florida, USA; b Emerging Pathogens Institute, University of Florida, Gainesville, Florida, USA; c Department of Environmental and Global Health, College of Public Health and Health Profession, University of Florida, Gainesville, Florida, USA; d Department of Medicine, College of Medicine, University of Florida, Gainesville, Florida, USA; McGill University

**Keywords:** cholera, evolution, Inaba, Ogawa, phylodynamics, serotype switch

## Abstract

Toxigenic Vibrio cholerae O1 serotype Ogawa was introduced involuntarily into Haiti in October 2010, and virtually all of the clinical strains isolated during the first 5 years of the epidemic were Ogawa. Inaba strains were identified intermittently prior to 2015, with diverse mutations resulting in a common phenotype. In 2015, the percentage of clinical infections due to the Inaba serotype began to rapidly increase, with Inaba supplanting Ogawa as the dominant serotype during the subsequent 4 years. We investigated the molecular basis of the serotype switch and confirmed that all Inaba strains had the same level of mRNA expression of the *wbeT* genes, as well as the same translation levels for the truncated WbeT proteins in the V. cholerae Inaba isolates. Neither *wbeT* gene expression levels, differential mutations, or truncation size of the WbeT proteins appeared to be responsible for the successful Inaba switch in 2015. Our phylodynamic analysis demonstrated that the V. cholerae Inaba strains in Haiti evolved directly from Ogawa strains and that a significant increase of diversifying selection at the population level occurred at the time of the Ogawa-Inaba switch. We conclude that the emergence of the Inaba serotype was driven by diversifying selection, independent of the mutational pattern in the *wbeT* gene.

**IMPORTANCE** Our phylodynamic analysis demonstrated that Vibrio cholerae Inaba strains in Haiti evolved directly from Ogawa strains. Our results support the hypothesis that after an initial Ogawa-dominated epidemic wave, V. cholerae Inaba was able to become the dominant strain thanks to a selective advantage driven by ongoing diversifying selection, independently from the mutational pattern in the *wbeT* gene.

## INTRODUCTION

Vibrio cholerae O1 exists as two major serotypes, namely, Ogawa and Inaba, that have a common O1 antigen referred to as the A-antigen in the cell wall lipopolysaccharide (LPS) (1–3). The difference between Ogawa and Inaba serotypes is determined by the methyl groups in the nonreducing terminal carbohydrate of the O1-antigen and is described as follows: the presence of the methylated LPS forming the B-antigen in Ogawa and the absence of methylated LPS resulting in the C-antigen in Inaba (1–6). The serotype switch is due to genetic alterations in the *wbeT* gene located in the *wbe* region of the V. cholerae O1 genome (2–4). This gene is also responsible for encoding a methyltransferase that catalyzes the methylation of the surface LPS (1–3, 5–7). Any deletion, or insertion, could cause a premature stop codon to hamper the methyltransferase that methylates the LPS, leading to the Inaba serotype (1, 3). Previous studies have demonstrated that LPS-specific immune responses are important in protective immunity to cholera (8). Therefore, it has been suggested that Ogawa-to-Inaba serotype switching during epidemic waves could be an effective evolutionary mechanism for V. cholerae to adapt in a host population, building immunity to the Ogawa serotype (9).

We have been continuously monitoring the cholera epidemic in Haiti by collecting and sequencing toxigenic V. cholerae O1 samples isolated from patients and the aquatic ecosystem since its onset in 2010 (3, 10–14). Five years into the epidemic, in 2015, a major shift occurred in the serotype of clinical isolates, with Ogawa, the initially dominant epidemic serotype, being replaced by serotype Inaba strains (3). As we have shown previously, the single-source introduction of V. cholerae O1 into Haiti offers a unique opportunity to study bacterial evolution in a naive population and characterize its population dynamic in a new ecological niche (14). In this study, we investigated the molecular basis and phylodynamics of the Ogawa-Inaba serotype switch using cutting-edge techniques, such as mass Western and Bayesian phylogeography, by taking advantage of a collection of toxigenic V. cholerae O1 genomic sequences obtained from 2010 through 2017.

## RESULTS

### Emergence of the Inaba serotype in Haiti Ouest Department with complete replacement of the Ogawa serotype.

**FIG 1:**
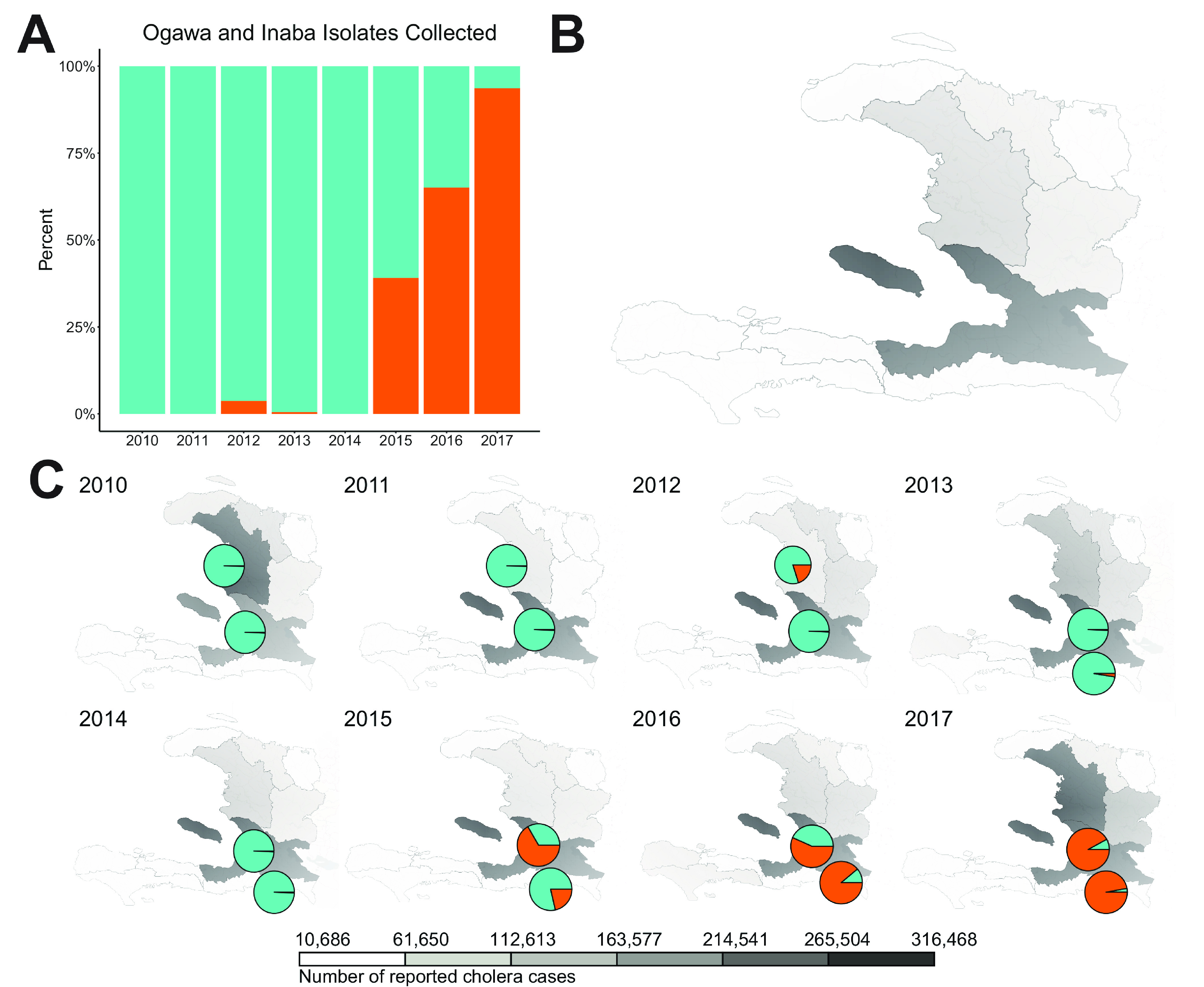
Number of cholera cases and isolates collected in Haiti from 2010 through 2017. (A) Proportion of Ogawa and Inaba isolates within the samples collected in Haiti from 2010 to 2018 and those for which the full genome was sequenced. (B) Map of cumulative cases of cholera in Haiti from 2010 to 2018 reported by PAHO. (C) Maps indicating the number of cholera cases in Haiti year by year from 2010 to 2017 that were reported by PAHO, and pie charts indicating the proportions for Ogawa (blue) and Inaba (orange) isolates from published isolates, as well as the proportions from the ones collected in this study (see Table S1 for details).

Our data set included full-genome sequences of toxigenic V. cholerae O1 isolates collected between 2010 and 2017 across the Artibonite region and the Ouest and Sud-Est departments (see Table S1 in the supplemental material). A yearly breakdown of the number of Ogawa and Inaba serotypes present in the samples collected during the V. cholerae epidemic in Haiti shows the increase in the Inaba serotype within the last 2 years of sampling (Fig. 1A). During the initial phases of the Haitian epidemic, all circulating V. cholerae O1 strains were serotype Ogawa. A few Inaba isolates were detected in 2012 and 2013, representing 4% and 1%, respectively, of the total V. cholerae population for those years. However, 5 years after its introduction in Haiti, the following unique characteristic began to emerge: the number of Inaba serotype strains collected in Haiti increased by 39%, steadily growing in number and becoming the predominant serotype in 2016 (65%), and coming close to replacing (94%) the Ogawa serotype in 2017 (Fig. 1A). The maps show the yearly burden in cholera cases across the departments in Haiti (Fig. 1B) and the proportions of serotypes per region of sampling (Fig. 1C). The Ogawa serotype was first introduced in the Artibonite region of Haiti, and it is here that we witness the first serotype conversions of 2012 and 2013 (Fig. 1C). V. cholerae spread quickly through the island, causing the most cholera cases in the Ouest Department, which is the region that includes Port-au-Prince (the capital and most populous city), with further extension into rural areas (Fig. 1B). The identification of the Inaba serotype in 2015 was not confined to a single region but affected both the Ouest and Sud-Est departments, suggesting either a founder event occurring and then spreading to the rest of the island or a series of serotype conversions due to convergent evolution occurring across the island at the same time.

**FIG 2:**
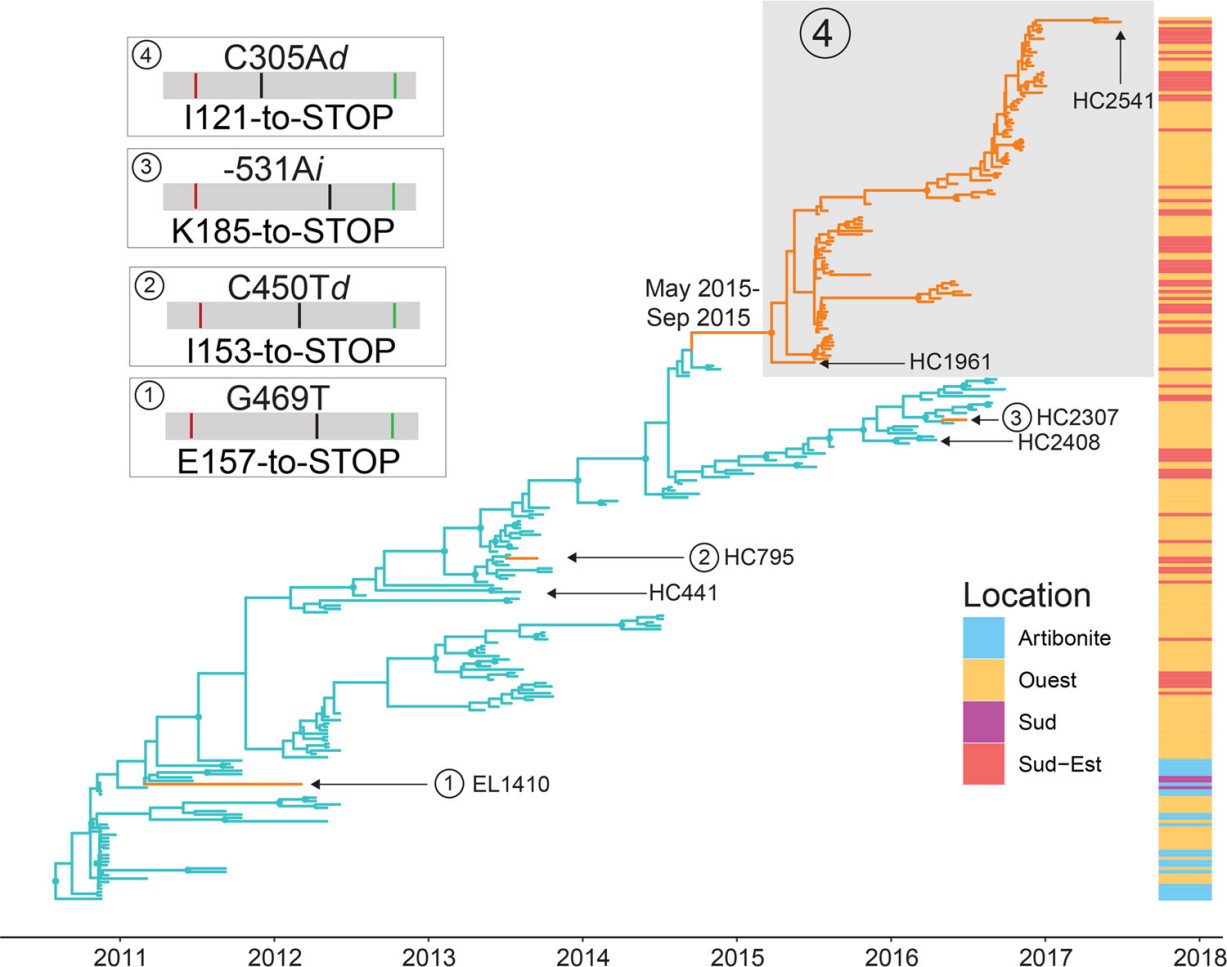
Contribution of toxigenic V. cholerae O1 isolates to the evolution of the cholera epidemic in Haiti between 2010 to 2018. MCC phylogeny for Ogawa and Inaba toxigenic V. cholerae O1 isolates collected between 2010 to 2018, inferred from genome-wide hqSNP data using the Bayesian phylogeography framework. Branch lengths are scaled in time by enforcing a relaxed molecular clock. Ogawa and Inaba states are indicated in blue and orange, respectively. Circles at internal nodes indicated high posterior probability (PP) support (PP, >0.9). The heatmap on the right of the tree indicates the department of origin for each tip of the phylogeny, with Artibonite in cyan, Ouest in yellow, Sud in purple, and Sud-Est in salmon. Ogawa and Inaba serotype isolates that were used for mass Western are shown with arrows. Circles numbered from 1 to 4 indicate the Inaba serotype isolates, and the boxes show the mutations in the *wbeT* gene and the result of the mutation. Within each box, the black line indicates the mutation and red and green lines indicate where the 5′ and 3′ mass Western peptide are (see Fig. 3).

To explore drivers for V. cholerae O1 serotype switching in Haiti, we employed a Bayesian phylogeography framework that allows examination of transitions between Ogawa and Inaba strains along internal (propagating lineages) and external (evolutionary dead end) branches of the tree (14). We inferred a Bayesian posterior distribution of phylogenies from 261 whole-genome bacterial strains sampled through the first 7 years of the Haitian epidemic (2010 to 2017) (Fig. 2), after testing for sufficient phylogenetic and temporal signal (see Fig. S1 in the supplemental material) and for the best fitting molecular clock and Bayesian demographic model priors (see Table S2 in the supplemental material). V. cholerae O1 Ogawa and Inaba serotype clades are clearly distinguishable in the maximum clade credibility (MCC) tree calculated from the posterior distribution of trees; the Ogawa serotype dominates the tree and its backbone until 2015, while a monophyletic clade of Inaba emerged in June 2015, with a 95% high posterior density interval (95% HPD) of May 2015 and September 2015 (Fig. 2). The mutation responsible for giving rise to the monophyletic Inaba lineage that successfully replaced the original Ogawa serotype was a deletion at nucleotide position 305 resulting in a premature stop codon at amino acid position 121 (Fig. 2). This mutation is shared by all the strains in the Inaba clade, namely, from its ancestor, sample HC1961 that was collected in the Cholera Treatment Center (CTC) of Gressier in the Ouest Department, to the last sample in our collection, sample HC2541 that was obtained in the CTC of Jacmel in the Sud-Est Department. Given the monophyletic nature of the Inaba clade, we hypothesize that the Inaba conversion was the result of one founder event, possibly occurring in the Ouest Department. Yet, convergent evolution occurred. Notably, three independent serotype switches occurred early the epidemic in 2012 (sample EL1410 from Artibonite) and in 2013 (sample HC795 from Sud-Est), as well as later in 2016 (sample HC2307 from Ouest) (Fig. 2). Although these samples present mutations that could cause a premature stop codon, these Inaba strains were not able to successfully spread across the host population and establish an Inaba serotype shift.

### The four different mutations in the *WbeT* gene present in the Inaba serotype strains have a similar effect on the expression of the *WbeT* gene and its product.

**FIG 3:**
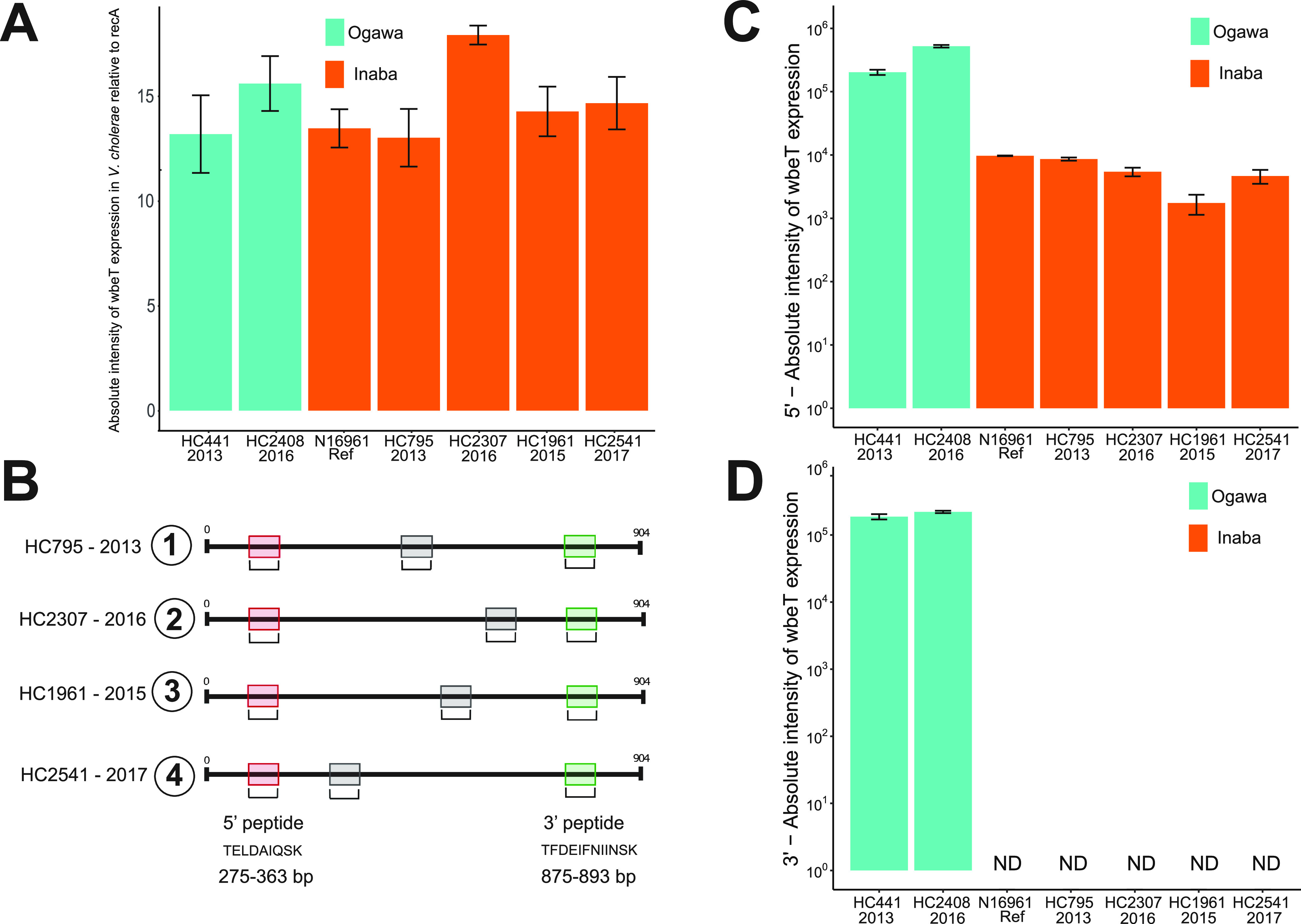
Quantitative expression and translation analysis of the *wbeT* gene on selected V. cholerae O1 samples collected in Haiti. (A) qPCR results showing the absolute intensity of *wbeT* expression (mRNA) relative to recA of Inaba and Ogawa strains. (B) The location of the peptides on the WbeT protein for mass Western quantification. Red displays the location of the 5′ peptide, and green displays the location of the 3′ peptide. The 5′ peptide was selected for its position before any mutations in *wbeT*, whereas the 3′ peptide was chosen for its position following a major mutation in *wbeT*. Essentially, 5′ and 3′ peptides of the N-terminal and C-terminal region, respectively, of WbeT were designed to detect and quantify the protein by mass spectrometry. (C) WbeT protein expression in regard to the 5′ peptide. (D) WbeT protein expression in regard to the 3′ peptide. Blue represents Ogawa, and orange represents the Inaba serotype.

We explored whether the effect of the mutation that successfully established the Inaba serotype in Haiti differed at the molecular level from the three earlier mutations that produced an occasional switch but did not lead to serotype replacement. To do so, we quantified the production of both *wbeT* mRNA and WbeT protein in four Inaba isolates in our collection chosen due to their unique mutations, as follows: HC795, HC2307, and HC1961, the first of the Inaba clade; and the most recent samples collected in the MCC phylogeny, HC2541 (Fig. 2 and 3). As a control, we also analyzed six Ogawa isolates phylogenetically linked to the Inaba serotypes; the Ogawa HC441 isolate was selected as phylogenetically closed to HC795 Inaba, and the same principle was applied for the Ogawa HC2408 isolate (linked to the HC2307 Inaba isolate) (Fig. 2 and 3). Results from real-time quantitative PCR (qPCR) showed that both Ogawa and Inaba isolates were expressing mRNA, with no substantial difference in expression (Fig. 3A). Next, we quantified the expressed WbeT protein from the same Inaba and Ogawa isolates employing mass Western, a mass spectrometry technique that allows protein quantification without the need of a specific antibody (see Materials and Methods) using probes designed to the 5′ and 3′ to detect expression and truncation, respectively (Fig. 3B). Ogawa HC441 and HC2408 isolates showed similar amounts of WbeT protein expression with the 5′ (Fig. 3C) and 3′ peptide (Fig. 3D), while, as expected (given the deletion in the *wbeT* gene), Inaba isolates showed a similar production of WbeT with the 5′ peptide (Fig. 3C) but not with the 3′ peptide (Fig. 3D). Both mRNA and protein expression levels of the *wbeT* gene oscillate among different Inaba strains that emerged over the years, and no clear pattern, potentially associated with increased (or decreased) fitness, could be observed. Therefore, we confirmed the expression of mRNA and translation of truncated WbeT proteins in each of the Inaba isolates that emerged in Haiti, suggesting that the successful spread of the lineage originated in 2015 appeared to be independent of the expression levels or size of truncation of *wbeT*.

### Selection analysis reveals ongoing diversifying selection in the Inaba serotype.

**FIG 4:**
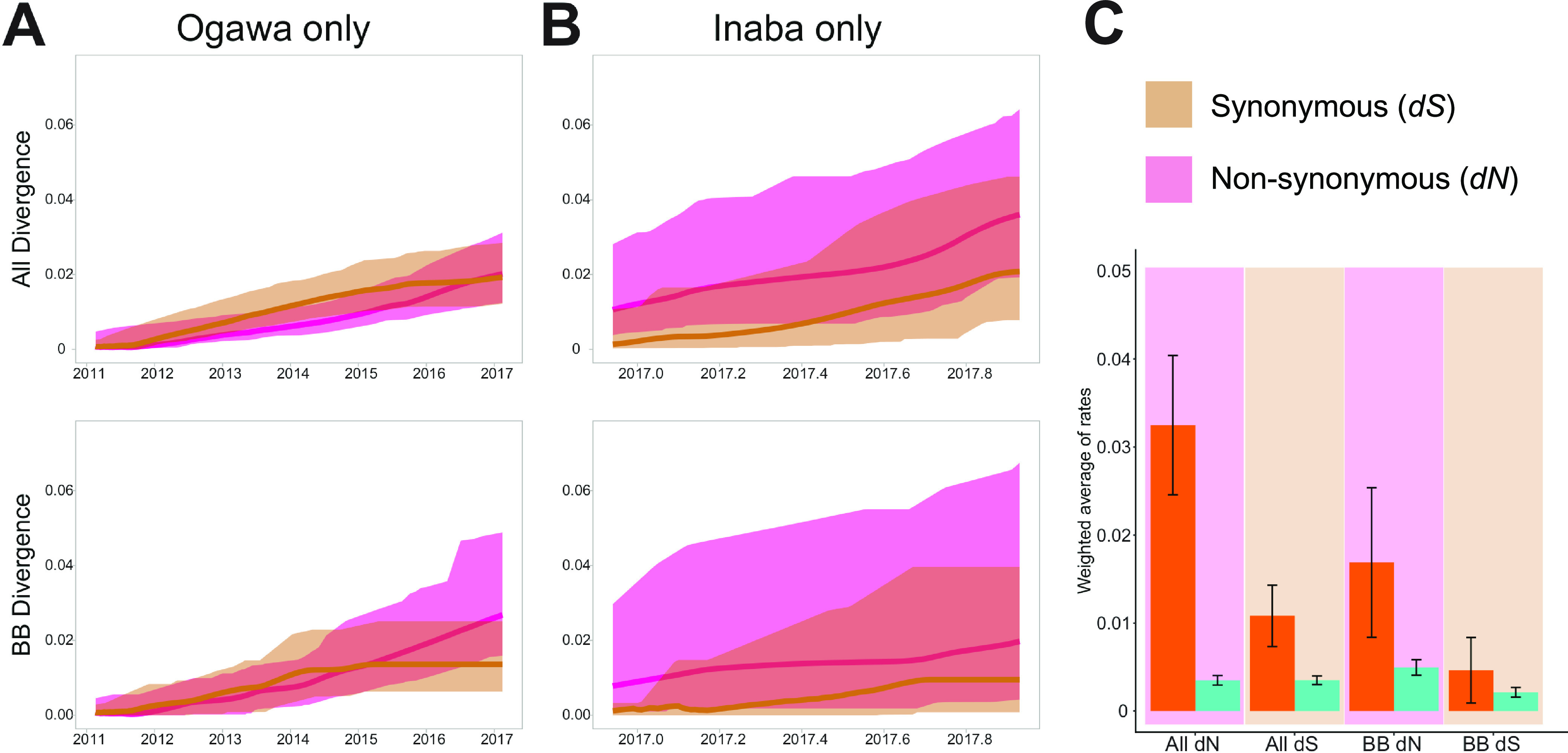
Divergence plots for both Ogawa (A) and Inaba (B) only. Tan represents the accumulation of synonymous mutations (*dS*), and pink represents the accumulation of nonsynonymous mutations (*dN*). (A) Ogawa-only divergence plots for all and backbone (BB) branches. (B) Inaba-only divergence plots for all and BB branches. (C) Weighted average of nonsynonymous and synonymous substitution rates for the Ogawa (blue) and Inaba (orange) serotype isolates. Both the Ogawa and Inaba data sets were based on 200 randomly sampled trees from the posterior distribution of molecular clock calibrated Bayesian phylogenies. All refers to an estimate based on all branches of the tree, while BB refers to estimates based on backbone branches of the phylogeny.

Next, we investigated whether the Ogawa-Inaba switch in Haiti was driven by diversifying selective pressure in the host population. We examined the dynamic changes in the accumulation of synonymous (*dS*) and nonsynonymous (*dN*) substitutions in whole cholera genomes across the phylogeny (Fig. 4; Fig. S2 in the supplemental material). An analysis of *dN* and *dS* rates in the data sets containing only Ogawa serotype strains (Fig. 4A) indicated that V. cholerae was driven mainly by genetic drift, after an initial phase of diversifying selection between 2011 and 2014, consistent with results from our previous study (14). On the other hand, during the successful spread of Inaba in Haiti, the Inaba-only data set shows *dN* rates consistently greater than *dS* rates (Fig. 4B), albeit with overlapping confidence intervals, which suggests ongoing diversifying selection. Therefore, we proceeded to estimate the nonsynonymous/synonymous substitution rate ratio (*dN/dS*) along the backbone path of the phylogeny, which represents the surviving lineage propagating through time. Overall, *dN/dS* was significantly greater than one (*dN*/*dS *= 3.0 *P < *0.01) in the portion of the tree dominated by Inaba strains (within the gray square in Fig. 3), while no significant difference was observed in the Ogawa portion of the phylogeny (Fig. 4C), thus indicating that the Inaba lineage that emerged in Haiti in mid-2015 (Fig. 2), and successfully displaced Ogawa strains (Fig. 1A), was characterized by ongoing diversifying selection.

## DISCUSSION

Cyclic outbreaks of Ogawa and/or Inaba have been reported previously where cholera is endemic (3–5, 7, 15). While the genetics of this serotype switch is known (mutations of the *wbeT* gene that inactivate the WbeT protein [1–4]), the evolutionary drivers of the serotype switch are nevertheless still unclear. A previous study reported serotype switching occurring randomly, with one serotype rarely taking over from the previous one in the endemic cholera population in Kolkata, India (5). Because both serotypes were dominating the study area at the same time, it is difficult to distinguish between an evolutionary-driven switch from an occasional random mutation (5). In the case of Haiti, the successful Inaba strain establishing the new epidemic wave appeared after 5 years of an Ogawa-dominated epidemic. During the Ogawa phase of the epidemic, we were able to document the occurrence of three isolated serotype switch events before the occurrence of a fourth that led to the serotype shift in the overall cholera population. Our mass Western and qPCR data showed that each switch was due to different mutations having the same biological effect as the fourth, suggesting that the type of mutation conferring serotype conversion does not grant a selective advantage to the serotype. Our data further suggest that the serotype Inaba emergence in Haiti was not linked to specific mutations within the *wbeT* gene. Instead, our phylodynamic analyses revealed that the serotype switch from Ogawa to Inaba was likely driven by diversifying selection at the population level.

While Ogawa and Inaba serotypes present similar severity and duration of illness (16), population-based studies have shown that persons initially infected with Ogawa strains show a greater degree of protection against subsequent Ogawa infection than against Inaba infection; in contrast, after an initial Inaba infection. there is equal protection against reinfection with both Inaba and Ogawa serotypes (17). Given these observations, it may be hypothesized that this difference in human immune response was a major driver in the serotype switch seen in 2015 in Haiti (9). However, at this time, no data on serotype-specific levels of immune response are available, leaving open the possibility that other factors were active, including possible strain-related differences in environmental survival. After a hiatus of 3 years with no reported clinical cases, toxigenic V. cholerae O1 serotype Ogawa has again emerged in epidemic form in Haiti beginning in September 2022. In work reported recently by our group, strains in this new epidemic shared a most recent common ancestor with a 2018 Haitian Ogawa strain isolated from the aquatic environment and clustered with the Ogawa clade that was circulating in 2015 to 2016 (18). It is likely that the re-emergence of epidemic disease was related, at least in part, to the waning of population immunity to infection with V. cholerae O1. However, it is unclear what role serotype-specific immunity may have played in this process, given the predominance of Inaba strains (which have been reported to provide comparable protection against infection with both Ogawa and Inaba serotype strains) in 2015 to 2019. The factors driving the emergence of this new epidemic wave remain to be determined; as further studies are conducted, we may also gain a better understanding of factors contributing to the replacement of Ogawa strains by Inaba in 2015 to 2019 reported in this paper.

## MATERIALS AND METHODS

### Ethics statement.

The study protocol was approved by the University of Florida Institutional Review Board (IRB) (number 201601821) and by Haitian National IRB.

### Sample collection, whole-genome mapping, and high-quality SNP (hqSNP) calling.

Between 2010 up to the end of 2017, approximately 800 toxigenic V. cholerae O1 strains were collected from patients with cholera who attended cholera treatment centers and clinics run by nongovernmental agencies and Haitian national clinics (3, 12–14). All V. cholerae isolates collected were confirmed by standard microbiological, biochemical, serological, and genetic analysis, and 261 strains from Haiti (154 Ogawa serotype strains and 107 Inaba ones; 116 from Mavian et al. (14), and 145 new samples from this study) (Table S1) were selected for high-quality full-genome next-generation sequencing (NGS) using in-house protocols described previously (10, 11, 13, 14). Bacterial genomic DNA extraction was performed as described previously (14). Sample library construction using the Nextera XT DNA library preparation kit was performed (Illumina). Whole-genome sequencing of all isolates was executed on the Illumina MiSeq platform for 500 cycles. SNPs were extracted and isolated as described in Mavian et al. (14). The SNP alignment in FASTA format was extracted from the VCF file from a custom python script (available upon request). The SNP alignment was filtered by site and annotated as described in Mavian et al. (14, 19). hqSNPs found in protein-coding regions of the V. cholerae O1 genome that were identified by annotation were used to produce a codon alignment that was extracted by in-house scripts (available upon request).

### Serotype classification and Sanger sequencing of isolates from Haiti.

Serotyping of V. cholerae O1 isolates was determined by agglutination assay, as described previously (3). Serotyping was confirmed by NGS, which resulted in 154 strains with the Ogawa serotype and 107 with the Inaba serotype by mapping mutations in the *wbeT* gene. Specifically, the *wbeT* gene sequence from each isolate, including the V. cholerae O1 N16961 strain, were extracted from the V. cholerae genome consensus sequence by mapping the reads to the *wbeT* reference (GenBank X58834). We also confirmed mutations in the *wbeT* gene, using a subset (*n* = 130 total, 89 Inaba and 41 Ogawa) of the Haitian strains, by amplicon-targeted PCR sequencing with Sanger (20). Extraction of genomic DNA was performed using the DNeasy blood and tissue kit (Qiagen, Inc.), and bacterial DNA was amplified with the following primers covering the entire *wbeT* gene: WF1 (5′-GATGTTCATGCGGTTTCCGT-3′) and WR1 (5′-CAGGAATTCACAGCACATCGC-3′). PCR was performed in a total volume of 50 μL containing NEBNext Q5 hot start PCR master mix (New England BioLabs, Ipswich, MA), 500 nM each primer, and 2.5 μg of genomic DNA. The following cycling conditions were used: initial denaturation at 98°C for 30 s; followed by 15 cycles or denaturation at 98°C for 10 s, annealing/extension at 65°C for 2 min; with a final extension at 65°C for 5 min. Direct Sanger sequencing of PCR products was then carried out as described previously (20).

### Map of Haiti displaying sample collection and serotype percentages.

Cumulative cholera cases in Haiti from 2010 to 2017 were obtained from the Pan American Health Organization (PAHO) (https://ais.paho.org/phip/viz/ed_colera_casesamericas.asp). Then cumulative cases from each year from 2010 up to 2018 were extracted from each department in Haiti, in order to show the number of cases each year in specific districts in Haiti. The python package Folium (https://python-visualization.github.io/folium/) was used to make maps, and an R script (available from the authors upon request) was used to plot the pie charts on the maps.

### Bayesian coalescent inference and discrete phylogeographic reconstruction.

Phylogenetic signal was determined using likelihood mapping test in IQ-TREE (21). TempEst was used to calculate the temporal signal of the maximum likelihood (ML) phylogeny and to plot the root-to-tip divergence (22). Bayesian phylogeography analysis (23), based on coalescence (24), was carried out with the BEAST v1.8.4 software package (25). The best molecular clock and demographic model was chosen by estimating the marginal likelihood of each model as described in Mavian et al. (14), with path sampling (PS) and stepping-stone (SS) methods, followed by Bayes Factor comparisons (25, 26) (Table S2). The Ogawa and Inaba serotype were used as character states for the Bayesian phylogeographic reconstruction. The maximum clade credibility (MCC) trees were obtained from the posterior distribution of trees with TreeAnnotator v1.8.4 after 10% burn-in (25). The MCC phylogeny was manipulated in R using the package GGTREE (27) for publishing purposes.

### Selection analysis.

Selective pressure driving the evolution of Ogawa and Inaba serotypes was assessed using a codon genome-wide alignment and a subset of 200 Bayesian MCC genealogies obtained randomly from the posterior distribution of trees for each subsampled data set. The weighted average of synonymous substitution rates (*K_S_*) and nonsynonymous substitution rates (*K_A_*) in the protein-coding regions of the V. cholerae O1 genome for all internal and external branches were obtained from a subset of 200 Bayesian MCC trees obtained randomly from the posterior distribution of trees, as described by Lemey et al. (28) and also implemented previously (14). The ratio of nonsynonymous (*dN*) and synonymous (*dS*) substitution rate (*dN/dS*), which provides information on whether evolution is occurring mainly through random genetic drift (*dN/dS*, ~1), positive selection (*dN/dS*, >1), or negative (*dN/dS*, <1) selection, was obtained from a subset of 200 Bayesian MCC trees obtained from the posterior distribution of trees, as described by Lemey et al. (28).

### RNA extraction, cDNA synthesis, and real-time PCR of *wbeT* mRNA.

All reagents were purchased from Thermo Fisher Scientific (Waltham, MA) unless otherwise indicated. Total RNA was isolated from samples using the RNeasy minikit (Qiagen, Germantown, MD). RNA was further concentrated using the Qiagen RNeasy MinElute clean-up kit and quantified using the Qubit RNA high-sensitivity (HS) assay kit. Five hundred nanograms of total RNA from each strain was used as the template in reverse transcriptase reactions. cDNA synthesis was performed using the SuperScript III first-strand synthesis system with random hexamers.

The primer sequences used to amplify *wbeT* cDNA were 5′-GATGCTGAAGGCGCAGAAATAG -3′ (*wbeT*_VC_634F) and 5′-AAACTCAGGATGTTCAGCGGAT-3′ (*wbeT_VC_*849R). Primers were also designed to amplify *recA* as the housekeeping gene. The SYBR green real-time PCR assay was performed in a 20-μL total volume containing SYBR select master mix (Applied Biosystems, Foster City, CA), 0.25 μM primers, and 2 μL of cDNA. Amplification of the primers, data acquisition, and relative analysis were carried out using QuantStudio 3 real-time PCR system.

### Mass Western of the WbeT protein in V. cholerae O1 samples from Haiti.

The 5′ peptide was selected for its position before any mutations in *wbeT*, whereas the 3′ peptide was chosen for its position following a major mutation in *wbeT* (Fig. 3A). Essentially, 5′ and 3′ peptides of the N-terminal and C-terminal region, respectively, of WbeT were designed to detect and quantify the protein by mass spectrometry. The isolates HC441, HC795, HC1961, HC2307, and HC2541 and then O39 and N16961 as controls were streaked onto a fresh LB plate from a frozen stock and allowed to incubate overnight at 37°C. A single colony was picked from the streak plate, allowed to grow in LB broth overnight shaking at 250 rpm, and pelleted at 16,000 × *g* for 1 min before being resuspended in 200 μL of cell lysis buffer (Cell Signaling, Danvers, MA) containing 1 mM sodium EDTA, 1 mM EGTA, 2.5 mM sodium pyrophosphate, 1 mM beta-glycerophosphate, 1 mM sodium orthovanadate, and 1 μg/mL leupeptin that was supplemented with 0.1 M phenylmethylsulfonyl fluoride (PMSF). Seven hundred micrograms of total protein from each isolate (listed above) was diluted in phosphate-buffered saline (PBS) and 4× Laemmli sample buffer supplemented with 10% beta-mercaptoethanol and loaded on a 12% mini-protean TGX precast SDS-PAGE gel (Bio-Rad, Hercules, CA) to run for 30 min at 300 V. The gel was stained with SimplyBlue SafeStain (Invitrogen, Carlsbad, CA) and destained. Protein bands corresponding to the expected size for WbeT were excised from a Coomassie-stained SDS-PAGE gel and then washed with 50 mM ammonium bicarbonate followed by dehydration with acetonitrile. The protein was reduced and alkylated in 45 mM dithiothreitol (55°C) and 100 mM chloroacetamide (in the dark at room temperature) for 45 min at each step, was washed with 50 mM ammonium bicarbonate, and dehydrated with acetonitrile. Following, the protein was dried in a speed vacuum to complete dryness before digesting overnight at 37°C with trypsin-ultra (New England BioLabs). The 5′ and 3′ peptides used for the identification of the WbeT protein by mass Western consist of a TELDAIQSK chain and a TFDEIFNIINSK chain of amino acids, respectively. Each peptide was extracted with 80% acetonitrile and 0.1% formic acid the next day, then cleaned up with ziptip (Millipore), and subjected to liquid chromatography-tandem mass spectrometry (LC-MS/MS) analysis. The TSQ Altis QQQ (Thermo Scientific) MS/MS system with ion funnel connected to a Vanquish Horizon ultra-high-performance liquid chromatography (LC) instrument (Thermo Scientific) was used to analyze the WbeT protein in different serotypes of cholera (The University of Florida, Interdisciplinary Center for Biotechnology Research [ICBR]-Proteomics and Mass Spectrometry Core). Prior to sample analysis, multiple reaction monitoring (MRM) parameters (precursor *m/z*, fragment *m/z*, radio frequency lens, and collision energy) for each unique peptide was optimized on the TSQ Altis QQQ MS using direct infusion of the authentic standards (synthesized by GenScript) at a concentration of 1 μg/mL in 50% acetonitrile containing 0.1% formic acid. Relative concentrations of the peptides were measured on an Accucore C_18_ column that was 2.1 by 100 mm and 2.6 μm (Thermo Scientific). A binary gradient of 0.1% formic acid in water and 0.1% formic acid in acetonitrile was used as mobile phases A and B, respectively. The LC gradient mobility was kept at 5% mobile phase B for the first 3 min and then diverted to waste. The gradient increased to 35% mobile phase B from 3 to 12 min, then increased from 35% to 40% mobile phase B from 12 to 14 min, then reached to 90% mobile phase B at 14.1 min, and held there until 18 min elapsed. It returned to 0% B in 0.1 min and then held at 0% B for 4 min at a flow rate of 0.2 mL/min. The mass spectrometer conditions were as follows: the spray voltage was applied at 3,500 V in the positive mode; and sheath gas, auxiliary gas, and sweep gas were set to 50, 10, 0, respectively. Ion transfer tube and vaporizer temperatures were set at 325°C and 100°C, respectively. For MRM monitoring, both Q1 and Q3 resolutions were set at 0.4 Da with the collision-induced dissociation (CID) gas set at 1.5 mTorr. The LC-MS/MS instrument was operated via Thermo Scientific Xcalibur Foundation software and acquired data processed for the generation of relative quantitation of the WbeT protein by the means of Quan Browser software.

### Data availability.

The genomic sequences have been deposited at NCBI Sequence Read Archive (SRA) under BioProject identifier (ID) PRJNA510624. All other data are available in the supplemental material. R and python scripts for the snp pipeline are available at the GitHub page https://github.com/salemilab/environmental_cholera_haiti.
