# Supplementary material for: Molecular Basis of the Toxigenic Vibrio cholerae O1 Serotype Switch from Ogawa to Inaba in Haiti
Source: Microbiol Spectr. 2022 Dec 20;11(1):e03624-22. doi: 10.1128/spectrum.03624-22 (PMC9927444; doi:10.1128/spectrum.03624-22)
Supplement: Supplemental file 1 — Fig. S1, legend of Table S1, and Table S2. Download spectrum.03624-22-s0001.pdf, PDF file, 0.1 MB [file spectrum.03624-22-s0001.pdf]

Supplementary figures and tables

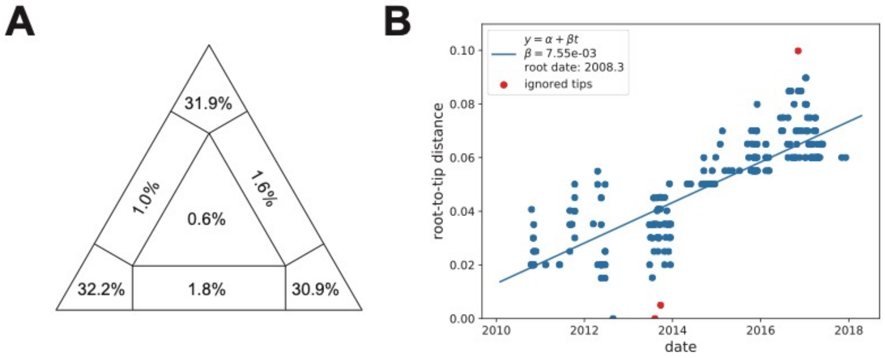

Figure S1. Estimation of phylogenetic signal and temporal signal for the *V. cholerae* O1 isolates isolated in Haiti. (A) Presence of phylogenetic signal was evaluated by likelihood mapping checking for alternative topologies (tips), unresolved quartets (center) and partly resolved quartets (edges) for each data set. (B) Linear regression of root-to-tip genetic distance within a maximum likelihood phylogeny against sampling time for each taxa. Temporal resolution was assessed using the slope of the regression, with positive slope indicating sufficient temporal signal. Correlation coefficient "r" are reported for each data set.

Table S1. List of Haitian V. cholerae O1 isolates used in this study. See excel file. The genomic sequences have been deposited into NCBI under Sequence Read Archive (SRA) under BioProject ID: PRJNA510624.

Table S2. Model selection of molecular clock and Bayesian demographic models to infer time-structured phylogeny for environmental *V. cholerae* O1 isolates collected between October 2010 and December 2017. Log marginal likelihood (log ml) values obtained by Stepping Stone (SS) and Path Sampling (PS), are reported for models that either used as priors: strict (SC) or uncorrelated relaxed lognormal (UCLN) molecular clocks, and constant (CONST), non-

parametric Bayesian skyline (BSP) or Gaussian Markov random field Bayesian skyride (GMRF) demographic models. Bayes Factor (BF) values are reported as ln(BF)ss and ln(BF)ps for PS and SS, respectively.

Model	log ml SS	Ln(BF)ss	log ml PS	Ln(BF)ps
**SC CONST**	-2353.6872	**9.6**	-2350.9015	**6.5**
**UCLN CONST**	-2344.1341		-2344.3745	
**SC BSP**	-2340.2384	**46.1**	-2337.1517	**46.2**
**UCLN BSP**	-2298.0155		-2298.2106	
**SC GMRF**	-2333.9708	**34.1**	-2330.111	**34.3**
**UCLN GMRF**	-2310.0551		-2310.1053	
**SC CONST**	-2353.6872	**13.4**	-2350.9015	**13.7**
**SC BSP**	-2340.2384		-2337.1517	
**SC CONST**	-2353.6872	**19.7**	-2350.9015	**20.8**
**SC GMRF**	-2333.9708		-2330.111	
**SC BSP**	-2340.2384	**6.3**	-2337.1517	**7.0**
**SC GMRF**	-2333.9708		-2330.111	
**UCLN CONST**	-2344.1341	**46.1**	-2344.3745	**46.2**
**UCLN BSP**	-2298.0155		-2298.2106	
**UCLN CONST**	-2344.1341	**34.1**	-2344.3745	**34.3**
**UCLN GMRF**	-2310.0551		-2310.1053	
**UCLN BSP**	-2298.0155	**-12.0**	-2298.2106	**-11.9**
**UCLN GMRF**	-2310.0551		-2310.1053	
